# Erythema Multiforme Induced by a “Milker's Nodule” Pseudocowpox Infection: A Case Report and Review of Literature

**DOI:** 10.1155/2021/5584773

**Published:** 2021-06-17

**Authors:** Kyle Wu, Sara de Menezes, Aaron Robinson

**Affiliations:** Department of Dermatology, St Vincent's Hospital Melbourne, 41 Victoria Parade, Fitzroy VIC 3065, Melbourne, Australia

## Abstract

Milker's nodule is caused by the pseudocowpox virus following inoculation from infected cattle. We report the case of erythema multiforme induced by pseudocowpox infection in an 18-year-old female from regional Australia. While erythema multiforme has been described as a complication of orf, it is rare as a sequela of pseudocowpox infection. Greater clinical knowledge of this disease and potential complications aid in guiding appropriate management of this phenomenon.

## 1. Introduction

Milker's nodule is a disease caused by pseudocowpox virus of the genus *Parapoxvirus* that is prevalent in non-metropolitan areas. The infection is of bovine source, typically involving infected teats and mouth of cattle. The disease typically manifests as localised erythematous-violaceous nodules at the site of inoculation after a brief incubation period of 5–15 days [1, 2]. Erythema multiforme (EM) is a hypersensitivity reaction of the skin or mucosa against specific antigens with ninety percent of cases induced by infections [3]. It is most commonly associated with herpetic infections, but scant reports have described EM reaction as a rare complication of pseudocowpox virus. We herein describe a case of pseudocowpox virus presenting with secondary erythema multiforme.

## 2. Case Description

An 18-year-old female from rural Australia with no medical history, presented to the emergency department with a three-day history of spreading, papulovesicular, pruritic eruption on bilateral knees, hands with preferential involvement of the right hand, as well as a superficial crusting of the lips. She reported being bitten on the right hand by her pet calf 2 weeks prior. She remained systemically well and denied constitutional symptoms including fevers or arthralgias. She denied any new or regular medications or preceding illness. She denied exposure to sheep, goats, gardening, or fish tanks.

**Figure 1:**
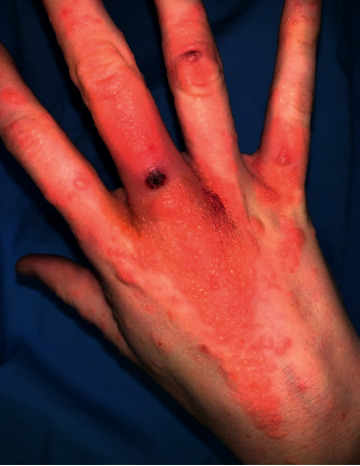
Dorsum of the right hand.

**Figure 2:**
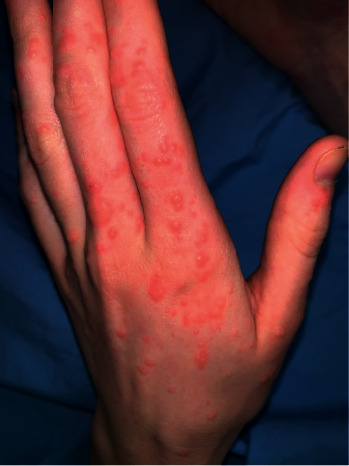
Dorsum of the left hand.

**Figure 3:**
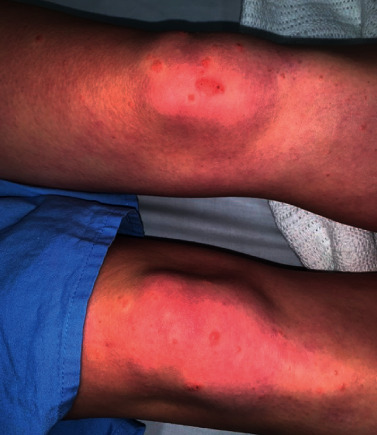
Bilateral knees.

**Figure 4:**
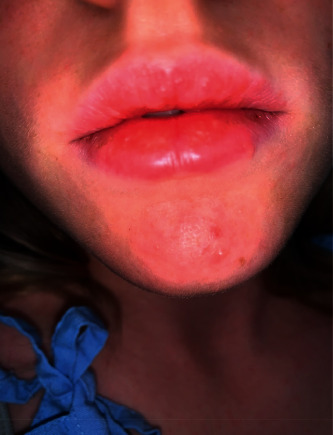
Superficial lip crusting.

On examination, there was a violaceous, eroded nodule on the dorsal aspect of her right middle finger with an associated erythematous-yellow papulovesicular eruption (Figure 1). Erythematous papules and nodules were present on the dorsal and palmar surfaces of her left hand (Figure 2) and on bilateral knees with no pustules (Figure 3). Superficial yellow crusting was present on the lips (Figure 4). There were no bullae observed and no lymphadenopathy.

Skin biopsy was non-specific, showing inflammatory material and keratin debris with no microorganisms noted on PAS, Grocott, Ziehl–Neelsen, and Wade Fite stains. PCR was positive for parapoxviruses. In combination with the history and examination findings, the patient was diagnosed with milker's nodule on the right hand with secondary erythema multiforme-like reaction involving bilateral upper and lower limbs with mucous membrane involvement. She was managed with betamethasone dipropionate ointment for pruritus; she was counselled on the use of gloves to reduce potential risk of transmission. On follow-up in one week's time, the eruption had completely resolved.

## 3. Discussion

Pseudocowpox, caused by zoonotic parapoxvirus, is generally transmitted by direct contact with infected lesions of cows, typically manifesting on patient's fingers and hands as milker's nodule. Erythema multiforme (EM) reaction, as presented in this case, is a rare complication of parapoxvirus infections. More common complications of milker's nodule include systemic symptoms of fever, lymphadenopathy, as well as possible bacterial superinfection. Bullous pemphigoid-like eruption has been reported as a rare sequela of orf and other parapoxviruses [4].

Despite similar clinical presentation, milker's nodule is a distinct entity from orf, the primary hosts of orf being sheep and goats rather than cattle [1, 5]. Other differential diagnoses aside from orf and other occupational zoonotic diseases include anthrax, atypical mycobacteriosis, tularaemia, and pyogenic granuloma [5]. Current PCR assays are unable to distinguish between pseudocowpox and orf with only subtle differences on tissue culturing [1]. Thus, the clinical history of suspected infection source and exposures is key to accurate diagnosis. Overall, milker's nodule and orf are notoriously underreported due to the typically self-limited natural history as well as common awareness amongst rural communities where it is most prevalent. Medical advice is often only sought once potential complications occur including EM [6].

The pathogenesis of EM is not completely understood, but hypothesized to be a cell-mediated immune reaction against specific antigens. In viral-associated EM, viral fragments are phagocytosed by Langerhans cells and transferred to epidermal keratinocytes, triggering the recruitment of CD4+ Th1 cells. Proinflammatory mediators such as IFN-*γ* are upregulated and induce an inflammatory cascade that promotes lysis of the infected keratinocytes and epidermal damage [4].

Viral triggers of EM include herpes simplex virus, Epstein–Barr virus, adenoviruses, enteroviruses, hepatitis viruses, influenza, and parapoxviruses [3]. While it is most commonly related to herpes simplex virus type 1, there are only scant reports describing EM reactions in pseudocowpox virus or milker's nodule [4, 7]. Within the *Parapoxvirus* genus, EM is again most commonly associated with orf infection rather than milker's nodule [4]. There are approximately 30 cases of orf-induced EM reported in the literature with reports of up to 18% of orf cases complicated by EM [8]. A Finish report from 1974 described an epidemic of milker's nodule with 7 out of 44 cases having secondary exanthem [9]. To our understanding, there has only been one other case of milker's nodule-associated EM reported in the English literature in an immunosuppressed patient triggered by graft-versus-host disease [6].

The paucity of literature also relates to the management of patients with secondary complications relating to milker's nodule. Systemic steroids have been demonstrated to be effective in treatment of patients with orf-associated EM [10]. Large lesions in immunocompromised patients have benefited from the use of cidofovir, an antiviral with broad-spectrum activity against DNA viruses [11]. In our case, no active treatment was necessary despite the complication of EM, with a complete resolution of disease within 3 weeks of initial inoculation. Supportive management and counselling still remain mainstays of treatment. It is important to emphasize the use of nonpermeable gloves when handling infected cattle to reduce risk of further infections. Misdiagnosis can lead to overtreatment including reported surgical excision of nodules and inappropriate antibiotic use [11]. Appropriate management is made possible with increased awareness and clinical knowledge of disease and its complications.

Milker's nodule is an underreported infection caused by the pseudocowpox virus of the *Parapoxvirus* genus, acquired from a bovine source. Targeted history taking and recognition of both common and rarer complications are critical in the diagnosis of milker's nodule. In addition to the classic local presentation, this case report illustrates the potential for secondary complications as a presenting complaint, including a rare systemic EM reaction. Accurate diagnosis of milker's nodule can help prevent exposing patients to unnecessary treatment.

## Data Availability

No data were used to support this study.
